# Conformational co-dependence between *Plasmodium berghei* LCCL proteins promotes complex formation and stability

**DOI:** 10.1016/j.molbiopara.2012.07.007

**Published:** 2012-10

**Authors:** Sadia Saeed, Annie Z. Tremp, Johannes T. Dessens

**Affiliations:** Department of Pathogen Molecular Biology, Faculty of Infectious and Tropical Diseases, London School of Hygiene & Tropical Medicine, Keppel Street, London WC1E 7HT, UK

**Keywords:** *Plasmodium berghei*, Crystalloid, GFP, Protein folding, LCCL domain

## Abstract

Malaria parasites express a conserved family of LCCL-lectin adhesive-like domain proteins (LAPs) that have essential functions in sporozoite transmission. In *Plasmodium falciparum* all six family members are expressed in gametocytes and form a multi-protein complex. Intriguingly, knockout of *P. falciparum* LCCL proteins adversely affects expression of other family members at protein, but not at mRNA level, a phenomenon termed co-dependent expression. Here, we investigate this in *Plasmodium berghei* by crossing a *Pb*LAP1 null mutant parasite with a parasite line expressing GFP-tagged *Pb*LAP3 that displays strong fluorescence in gametocytes. Selected and validated double mutants show normal synthesis and subcellular localization of *Pb*LAP3::GFP. However, GFP-based fluorescence is dramatically reduced without *Pb*LAP1 present, indicating that *Pb*LAP1 and *Pb*LAP3 interact. Moreover, absence of *Pb*LAP1 markedly reduces the half-life of *Pb*LAP3, consistent with a scenario of misfolding. These findings unveil a potential mechanism of conformational interdependence that facilitates assembly and stability of the functional LCCL protein complex.

## Graphical abstract

**Graphical abstract:**

This paper identifies conformational codependence between *Plasmodium berghei* LCCL proteins by crossing *Pb*LAP1-KO and *Pb*LAP3/GFP parasite lines.

**Figure:**
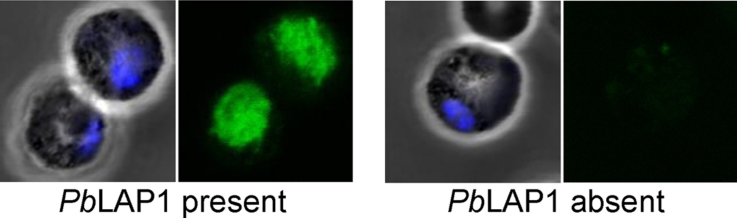

**Highlight:**

► Co-dependent expression of LCCL proteins appears absent in *Plasmodium berghei*. ► However, *P. berghei* LCCL proteins are conformationally co-dependent. ► This mechanism promotes LCCL protein complex formation and stability. ► Conformational co-dependence could be the mechanism behind co-dependent expression.

The LCCL protein family of malaria parasites is a group of six highly conserved and structurally related proteins that possess a modular architecture with multiple domains implicated in protein, lipid and carbohydrate binding [1–5]. The family is named after the *Limulus* clotting factor C, Coch-5b2, Lgl1 (LCCL) domain [6] which is found in all except one family member. LCCL proteins possess a consensus endoplasmic reticulum (ER) signal peptide at the amino-terminus, but other known organelle targeting sequences are absent [1,4]. The LCCL protein family members are referred to as *Plasmodium* LCCL domain-containing proteins (*P*CCp) [4] used mostly for *Plasmodium falciparum*, and *Plasmodium* LCCL-lectin adhesive-like proteins (*P*LAP) mostly used in the context of *Plasmodium berghei* [5]. In *P. berghei*, all of the family members except *Pb*LAP3 have been studied by gene disruption, which has revealed that they have very similar loss-of-function phenotypes characterized by a loss of sporozoite development in the oocyst and concomitant loss of sporozoite transmission [1,7–9]. Conversely, knockout of *Pf*CCp2 and *Pf*CCp3 expression in *P. falciparum* does not appear to affect sporozoite development, but blocks the transition from midgut to salivary gland sporozoites [4]. *Plasmodium* LCCL proteins thus play vital roles in sporozoite transmission.

There is a clear consensus that all *P. berghei* LCCL protein family members are expressed in gametocytes based on a combination of GFP reporter studies and GFP-tagging experiments [7,10–12]. This fully agrees with the reported expression of the LCCL protein family members in *P. falciparum* as determined by immunofluorescence and immunoblot [4,13–16]. The similar expression patterns and the highly similar loss-of-function phenotypes of the *Pb*LAPs strongly indicate that these molecules are functionally interdependent, are involved in the same molecular processes in the parasite, and operate as a protein complex. This notion is further supported by the observation that several *Pb*LAPs are targeted to the crystalloid organelle in the downstream ookinete stage [7,11]. Indeed, molecular interactions between *P. falciparum* LCCL protein family members have been shown in co-immunoprecipitation experiments using gametocytes [16], providing evidence for protein complex formation.

Another line of evidence that supports a functional relationship between the LCCL protein family members is provided by observations in *P. falciparum* that *Pf*CCp proteins are co-dependently expressed [14,16]. These studies showed using immunofluorescence and immunoblotting that genetic disruption of a single *pfccp* gene not only abolishes expression of its cognate gene product as expected, but also reduces or abolishes the expression of other LCCL proteins. Moreover, this process occurs at the protein, but not at the transcript level. It remains unclear what the molecular mechanisms are that underlie this phenomenon. In this study we investigated whether co-dependent expression exists in *P. berghei*. To this purpose we generated a double mutant parasite line by crossing a structural gene knockout parasite line for *Pb*LAP1 (also known as *Pb*SR; PlasmoDB ID: PBANKA_103520), named *Pb*SR-KO [7], with a mutant parasite line expressing full-length *Pb*LAP3 (PlasmoDB ID: PBANKA_020450) tagged at the carboxy-terminus with GFP, named *Pb*LAP3/GFP [11]. *Pb*SR-KO parasites display the typical null mutant phenotype of all the *Pb*LAP proteins, characterised by a loss of sporozoite development and transmission [7]. This parasite line does not exhibit GFP-based fluorescence. By contrast, parasite line *Pb*LAP3/GFP exhibits strong GFP fluorescence in gametocytes and displays a wildtype phenotype throughout the life cycle [11]. The two parasite lines were crossed by culturing ookinetes from mixed gametocyte populations, followed by feeding the resulting ookinetes to *Anopheles stephensi* vector mosquitoes in membrane feeders. Because the *pblap* alleles are maternally inherited [9], cross-fertilization events between female gametes of *Pb*LAP3/GFP and male gametes of *Pb*SR-KO parasites should give rise to infective sporozoites. At three weeks post-infection, sporozoites were transmitted to naïve mice by infected mosquito bites, and double mutants containing both of the modified *pblap* alleles were selected from the resulting patent blood stage infection by drug selection followed by limiting dilution cloning, as described [17]. The presence of the two modified *pblap* alleles, as well as the absence of the equivalent unmodified alleles, was confirmed by diagnostic PCR. A ca. 1.1 kb fragment specific for the modified *pblap1* allele was amplified from the double mutant and parental *Pb*SR-KO parasite line (Fig. 1A). Similarly, a ca. 1.8 kb fragment specific for the modified *pblap3* allele was amplified from both the double mutant and parental *Pb*LAP3/GFP parasite line (Fig. 1A). In addition, fragments of ca. 1.4 kb and 0.8 kb diagnostic for the wildtype *pblap1* and *pblap3* alleles, respectively, were absent from the double mutant parasites (Fig. 1A). The presence of the *pblap3::gfp* allele in the double mutant was further verified by Southern analysis: a *pblap3*-specific probe detected a 3.4 kb fragment in *Hin*dIII-digested genomic DNA of the *Pb*SR-KO parental line, but a 9.5 kb fragment in the *Pb*LAP3/GFP parental line, as expected (Fig. 1B and C). A 9.5 kb fragment corresponding to the modified *pblap3* allele was also detected in the double mutant, while the 3.4 kb fragment corresponding to the wild-type *pblap3* allele was absent in this parasite (Fig. 1B). Similarly, a *hdhfr*-specific probe detected a 9.5 kb fragment in both the *Pb*LAP3/GFP parent and double mutant, and no signal in the *Pb*SR-KO parent, as expected (Fig. 1B and C).

Validated clones of the double mutant parasite line were assessed by western blot analysis for the expression of the *Pb*LAP3::GFP fusion protein in gametocytes, in comparison with the parental lines. This showed normal expression of the protein in question (Fig. 2A), demonstrating that knockout of *Pb*LAP1 had not adversely affected expression levels of *Pb*LAP3 as is the case for the orthologous proteins in *P. falciparum* [16]. This result indicated that gametocyte stage co-dependent expression does not occur in *P. berghei*. Surprisingly, however, GFP fluorescence levels in gametocytes of the double mutant parasite line were dramatically reduced compared to the parental *Pb*LAP3/GFP gametocytes (Fig. 2B), indicating that the absence of *Pb*LAP1 has affected the ability of the *Pb*LAP3::GFP chimera to generate fluorescence. To further investigate the expression and subcellular distribution of *Pb*LAP3::GFP in gametocytes of the double mutant parasite line we performed immunofluorescence using commercially available anti-GFP antibody. Gametocytes were purified as described [18], fixed for 20 min in 4% paraformaldehyde, washed twice with PBS, blocked and permeabilized for 1 h in PBS supplemented with 0.1% Triton X-100 and 1% BSA. Then the gametocytes were labelled with anti-GFP antibodies conjugated to FITC (ab65180, Abcam, diluted 1 in 1000) for 1 h at room temperature. After a further two PBS washes the gametocyte suspension was examined by confocal microscopy. FITC signal in the double mutant gametocytes was clearly detectable compared to GFP-negative wildtype parasite controls (Fig. 2C), consistent with expression of the *Pb*LAP3::GFP fusion protein in the double mutant. Moreover, the subcellular distribution of FITC signal (Fig. 2C) was comparable to that of GFP signal in the parental *Pb*LAP3/GFP parasite line (Fig. 2B), demonstrating that the subcellular distribution of *Pb*LAP3::GFP had not drastically changed in the absence of *Pb*LAP1. It is therefore unlikely that a change in the protein's localization to a subcellular compartment less favourable for generating GFP fluorescence is responsible for the loss of GFP fluorescence in the double mutant. We postulate that a more likely explanation for the loss of GFP fluorescence is based on a *Pb*LAP1–*Pb*LAP3 molecular interaction: the imposed loss of this interaction in the double mutant parasite line causes a conformational change in *Pb*LAP3 that is adverse to functionality of its GFP tag. Indeed, the folding of amino-terminal fusions with GFP has long been known to influence fluorescence levels of chimeric GFP [19].

The notion that in the absence of *Pb*LAP1 the *Pb*LAP3::GFP fusion protein adopts a different conformation and hence is in a potential state of ‘misfolding’ led us to investigate the stability of the protein post-synthesis by western blot. For this purpose ookinete cultures were set up from gametocytemic blood as described [20] and after 24 h ookinetes were purified and analysed. Western blot revealed that considerably less *Pb*LAP3::GFP was present in the double mutant than in the parental *Pb*LAP3/GFP ookinete population, relative to the *P. berghei* ookinete-specific CS and TRAP related protein (*Pb*CTRP) [21] (Fig. 2D). In fact, the double mutant ookinetes possessed only 16% of the *Pb*LAP3::GFP level of the parental *Pb*LAP3/GFP line as determined by ImageJ analysis as recommended [http://rsbweb.nih.gov/ij] and normalized against *Pb*CTRP levels. This implies that *Pb*LAP3 is markedly less stable in the absence of *Pb*LAP1 than in its presence, a scenario that is consistent with a misfolded protein being actively removed from the cell.

Our combined findings unveil a mechanism of conformational interdependence between *Pb*LAP1 and *Pb*LAP3 that stabilizes the molecules when they interact and as such promotes the assembly and durability of the functional LCCL protein complex. It is highly plausible that such a mechanism extends to the other LCCL protein family members. The fact that all *Pb*LAPs studied so far have exhibited very similar loss-of-function phenotypes strongly points to a functional interdependence of these molecules, which is obviously compatible with a scenario in which assembly of a functional LCCL protein complex is prevented when individual components are lacking, as is the case in *Pb*LAP knockout parasites. A similar system of conformational interdependence, if it were present in *P. falciparum*, could provide the underlying mechanism for the observed co-dependent expression of its LCCL proteins in gametocytes. Gametocytogenesis in *P. falciparum* takes considerably longer than in *P. berghei* and, consequently, much more time is available for any misfolded LCCL proteins to be degraded and removed before the gametocytes reach maturity, especially as the *PfCCp* proteins are already expressed early during gametocytogenesis [15]. Moreover, the varying degrees of co-dependent expression observed in *P. falciparum* between different family members [16] could reflect quantitative differences in their dependence on each other to fold correctly.

## Figures and Tables

**Fig. 1:**
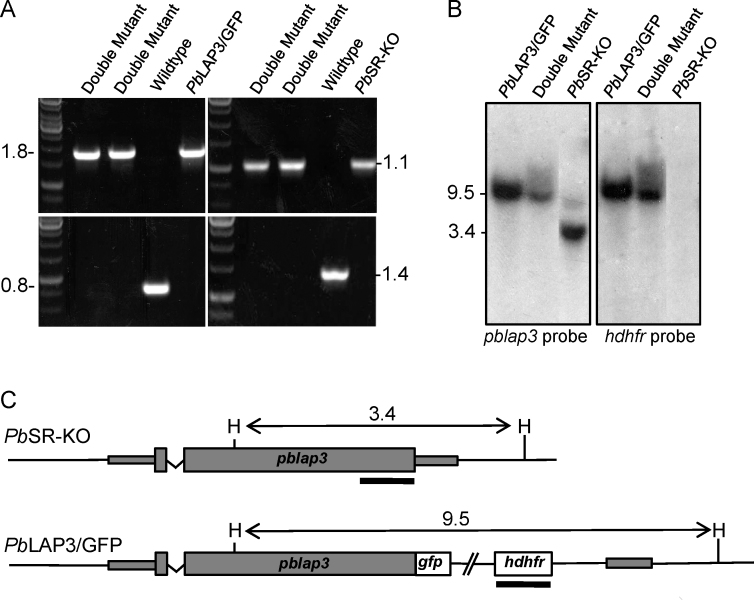
Molecular analyses of the parental parasite lines *Pb*SR-KO and *Pb*LAP3/GFP, and double mutant parasites derived from a *Pb*SR-KO × *Pb*LAP3/GFP genetic cross. (A) PCR diagnostic for the disrupted *pblap1* allele (top right panel; primers [CGCGATG ACCCCCAAGAGGGG] and [CGCCTTCACGCTGATGT]); the GFP-tagged *pblap3* allele (top left panel; primers [ACAAAGAATTCATGGTTGGTTCGCTAAACT] and [CCTCAAGATAGTTACGAATTTAAC]); the wildtype *pblap1* allele (bottom right panel; primers [CATAATATGCATCTAGAACCAACTTTTC] and [AACGGGATCTTCTAGAATTTAATATAAGCGTTTCAAAAAGGTAAATG]); and the wildtype *pblap3* allele (bottom left panel; primers [ACGAAGTTATCAGT CGAGGTACCTAGCGGAAACAACAATGTTC] and [CCTCAAGATAGTTACGAATTTAAC]). B) Southern blot analysis of *Hin*dIII-digested genomic DNA using a *pblap3*-specific probe (left panel) and a *hdhfr*-specific probe (right panel). (C) Schematic diagram showing the structure of the *pblap3* alleles in parasite lines *Pb*SR-KO and *Pb*LAP3/GFP. Indicated are the *Hin*dIII restriction sites (H), sizes of the predicted *Hin*dIII restriction fragments, and regions corresponding to probes used in B (thick lines).

**Fig. 2:**
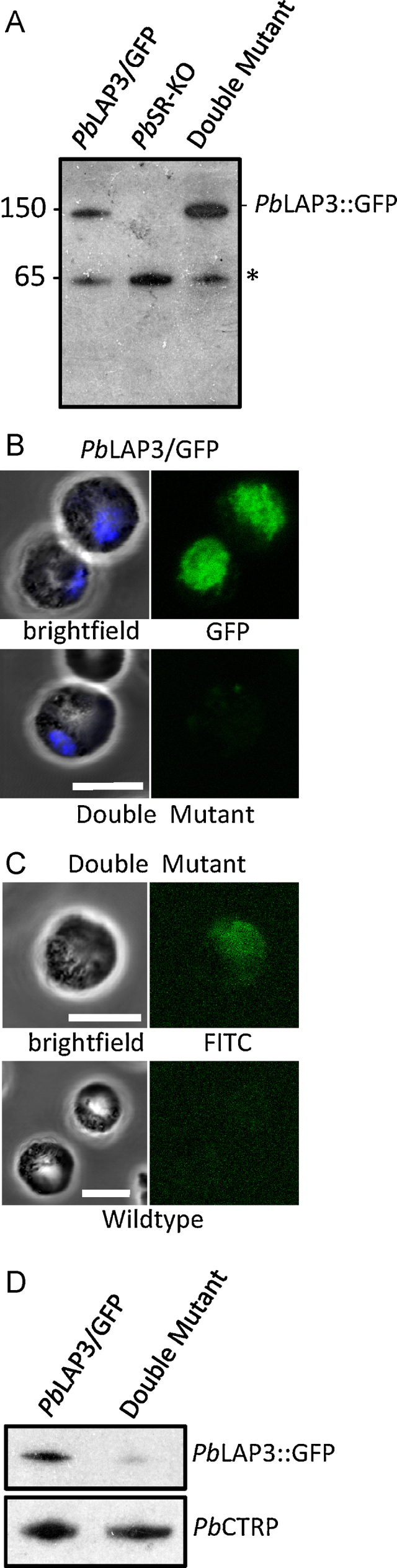
Expression and distribution of the *Pb*LAP3::GFP fusion protein in the parental parasite lines *Pb*SR-KO and *Pb*LAP3/GFP, and double mutant parasites derived from a *Pb*SR-KO × *Pb*LAP3/GFP genetic cross. (A) Western blot of purified gametocyte samples of parasite lines *Pb*SR-KO, *Pb*LAP3/GFP, and the double mutant, using anti-GFP antibodies. The blot shows bands of ca. 150 kDa corresponding to the *Pb*LAP3::GFP chimera, and of ca. 65 kDa (*) corresponding to a non-specific protein that cross reacts with the antibody. (B) Confocal GFP fluorescence images of macrogametocytes of parasite line *Pb*LAP3/GFP and the double mutant parasite. GFP images were taken with the same gain settings. Nuclei were stained with Hoechst (blue). Bar = 5 μm. (C) Confocal FITC immunofluorescence images of macrogametocytes of double mutant and wildtype parasites. FITC images were taken with the same gain settings. Bar = 5 μm. (D) Western blot of purified ookinete samples of parasite lines *Pb*LAP3/GFP and the double mutant showing *Pb*LAP3::GFP fusion protein relative to the ookinete loading control *Pb*CTRP. (For interpretation of the references to color in this figure caption, the reader is referred to the web version of the article.)
